# Transabdominal Preperitoneal Repair Versus Lichtenstein’s Open Hernia Repair for Inguinal Hernias: A Retrospective Study of 120 Cases

**DOI:** 10.7759/cureus.81804

**Published:** 2025-04-06

**Authors:** Supriya Bhondve, Kashif F Ansari, Rajalakshmi Venkateswaran, Balakrishan Menon, Ajay H Bhandarwar, Snehal M Dandge, Ravi A Landge

**Affiliations:** 1 General Surgery, Grant Government Medical College and Sir JJ Group of Hospitals, Mumbai, IND

**Keywords:** intraoperative outcomes, lichtenstein's open hernia repair, perioperative outcomes, transabdominal preperitoneal repair, visual analogue scale

## Abstract

Introduction: Inguinal hernia repair is one of India's most common procedures in general surgery. The advent of minimally invasive surgery for this condition has revolutionized its treatment. This study compares the outcomes of transabdominal preperitoneal (TAPP) repair and Lichtenstein’s open hernia repair, focusing on specific intraoperative and perioperative outcomes.

Materials and methods: A retrospective analysis was conducted on patients who underwent either TAPP repair or open hernia repair at a single tertiary care center between June 2021 and June 2024. A total of 120 patients were included, with Group A comprising 60 patients who underwent TAPP repair and Group B comprising 60 patients who underwent open hernia repair. In addition to demographic data, parameters such as operative time, length of hospital stay, postoperative pain score using the Visual Analogue Scale (VAS), and incidence of postoperative complications were analyzed. Statistical comparisons were made using chi-square and t-tests, with a significance level set at p < 0.05.

Results: The mean operative time for Group A and Group B was 137.43 ± 24.41 minutes and 108.91 ± 36.73 minutes, respectively, which was statistically significant (p < 0.001). Although individual complications varied, the complications were 11.66% (seven patients) in Group A and 38.33% (23 patients) in Group B. The VAS revealed that the average pain score at 24-48 hours was 4.05 ± 0.80 in Group A and 4.3 ± 0.74 in Group B, indicating a statistically significant lower pain level in Group A (p = 0.03). At the end of one week, the average pain score was significantly lower in Group A (1.18 ± 0.42 vs. 1.55 ± 0.67, p < 0.001). The average duration of hospital stay was 2.3 ± 0.64 days for Group A and 3.01 ± 0.911 days for Group B. An unpaired t-test showed statistically significant differences between the two groups' hospital stay duration and time taken to return to normal work (t = 4.98, p < 0.001 and t = 14.041, p < 0.001, respectively). The average number of days for which analgesics were required was 1.1 in Group A and 1.6 in Group B.

Conclusions: TAPP repair offers significant advantages in terms of postoperative pain and recovery time compared to traditional open hernia repair. Although TAPP repair requires a longer operative time, its benefits, such as reduced recovery duration and lower postoperative pain, could lead to better patient outcomes and reduced strain on healthcare resources. Both techniques showed comparable complications and hernia recurrence rates, suggesting that TAPP repair is a safe and effective alternative to open hernia repair. Future studies with larger sample sizes and longer follow-up periods are recommended to further assess the long-term efficacy and cost-effectiveness of TAPP repair compared to open hernia repair.

## Introduction

The word hernia is derived from a Latin term meaning "rupture." It is a condition characterized by the abnormal bulging of contents from the abdominal cavity through a weakness in the wall that contains them. The prevalence of inguinal hernia in India is estimated to be 1.5-2 million cases per year [1], making inguinal hernia repair one of the most common elective surgeries performed in the country.

Lichtenstein’s open hernia repair has long been preferred among most surgeons. Many still consider it the optimal approach due to its comparable recurrence rates with laparoscopic hernia repair [2] and its shorter learning curve [3,4]. Studies have shown that a minimum of 60 open hernia repair surgeries [3] is required to achieve proficiency. In contrast, approximately 100 laparoscopic hernia repair surgeries are needed to reach the same level of competence [4].

Just as mesh repair has replaced tissue repair, laparoscopic hernia repair has gained significant popularity among surgeons. The laparoscopic technique is similar to the open preperitoneal approach and can be performed either transabdominally or via a totally extraperitoneal approach [5].

The present study compares treatment groups that have undergone laparoscopic repair of inguinal hernia using the transabdominal preperitoneal (TAPP) repair and those who underwent open hernia repair, with respect to certain intraoperative and postoperative parameters.

## Materials and methods

The study is a retrospective comparative study conducted at a tertiary care center in Mumbai from June 2021 to June 2024. A total of 120 male patients between the ages of 18 and 65 years, each with a unilateral inguinal hernia (direct or indirect), were included in the study. The hospital records of these patients were analyzed for data collection. Patients diagnosed with recurrence, irreducible or strangulated hernias, and bilateral inguinal hernias were excluded from the study.

All patients in the study group were operated on by consultant surgeons. This study compared two techniques of hernia repair: Lichtenstein’s open hernia repair and TAPP repair. Group A (n = 60) comprised patients who underwent TAPP repair, and Group B (n = 60) comprised those who underwent open hernia repair. Being a retrospective study, randomization was found to have been done based on the patient's preferences and pre-anesthetic fitness for either procedure.

Data collected from the patients' records included operative time, incidence of immediate postoperative complications (such as seroma, hematoma, surgical site infection, cord hematoma, testicular pain, and urinary retention), postoperative pain score, incidence of chronic neuralgia, number of days analgesia was required immediately following surgery, and time taken to return to their preoperative normal work life. Postoperative pain perception was documented from scores marked on the Visual Analogue Scale (VAS) charts in each of their records at 24-48 hours, at one week, and at one month postoperatively.

Operative techniques

Transabdominal Preperitoneal Repair

**Figure 1:**
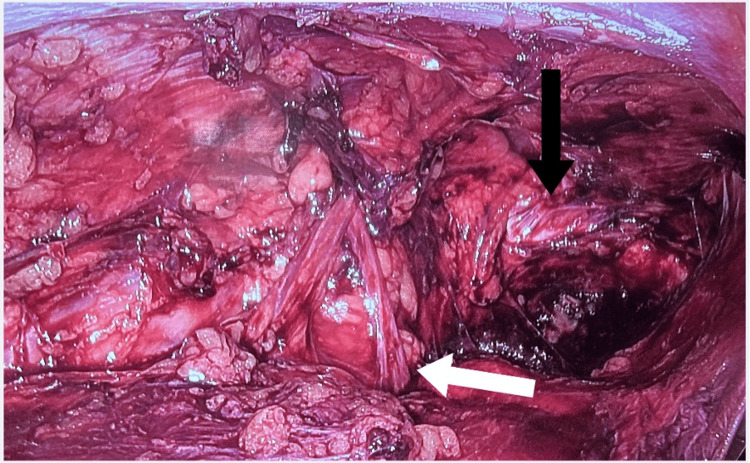
TAPP repair procedure, depicting the important structures in the preperitoneal. The white arrow shows the spermatic cord structures, and the black arrow indicates the Cooper’s ligament TAPP: transabdominal preperitoneal

The procedure was performed under general anesthesia with the patient in the supine position. The surgeon operated from the head end of the patient. A 10 mm camera port was inserted supra-umbilically. Two 5 mm ports were inserted on either side of the umbilicus, 5 cm away from it, with the working port on the side of the hernia positioned 2 cm higher. Pneumoperitoneum was created using carbon dioxide. A peritoneal flap was created 5 cm proximal to the anterior superior iliac spine level and extended horizontally to the medial umbilical ligament. Dissection of the Retzius space was performed to expose Cooper’s ligament medially, and the space of Bogros was dissected until the psoas muscle was visualized. The hernia sac was then reduced. It was dissected from the defect and separated from the cord structures. Adequate parietalization of the cord structures was performed until they were free from attachments to the peritoneum (Figure 1). After visualization of the myopectineal orifice, a polypropylene mesh was placed in the preperitoneal plane, covering all hernial orifices. The peritoneal flap was then closed.

Lichtenstein’s Open Hernia Repair

**Figure 2:**
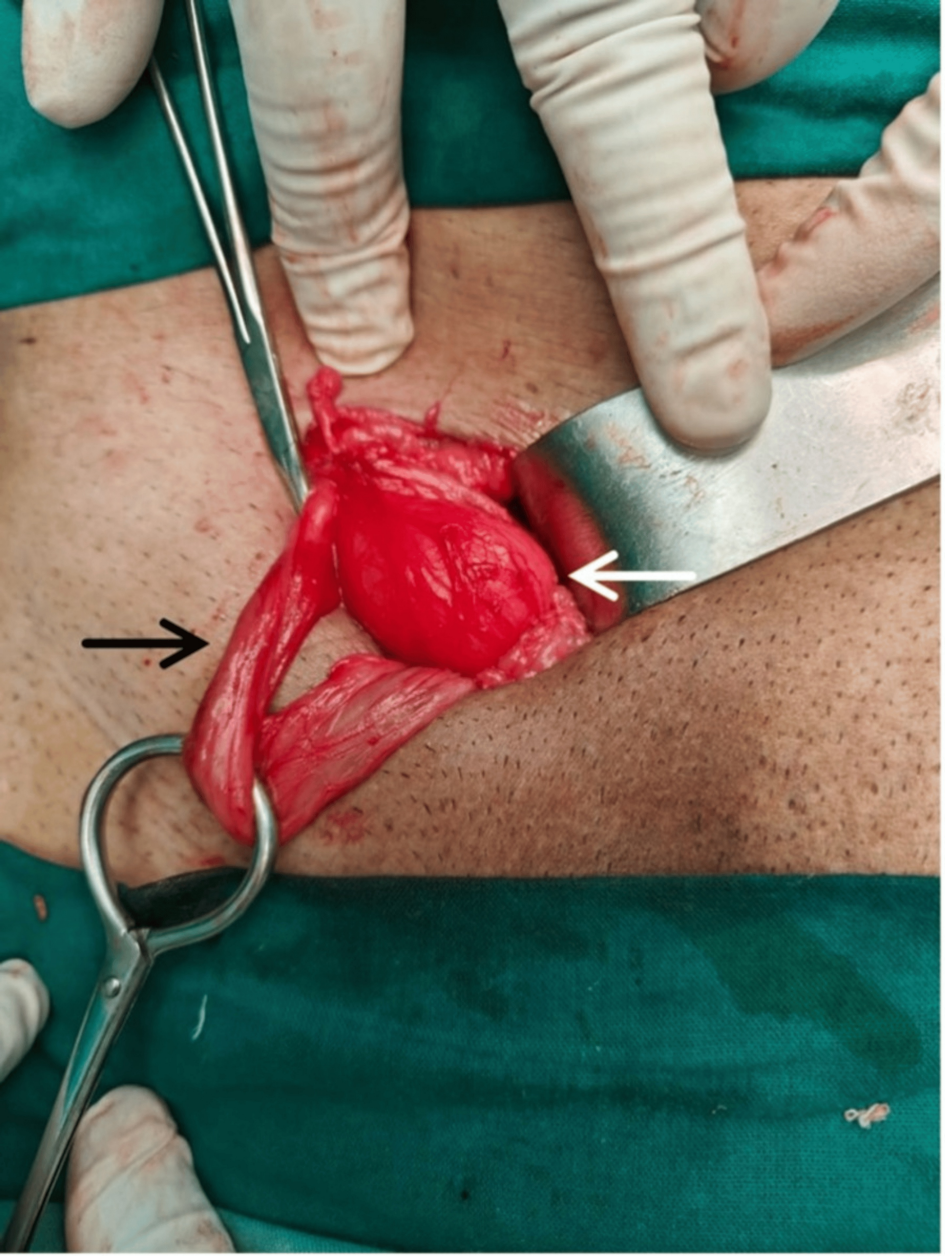
Lichtenstein's open hernia repair surgery with the image showing the hernia sac and the cord structures delineated from it. The white arrow indicates the hernia sac, and the black arrow indicates the cord structures

The open hernia repair was performed under spinal anesthesia with the patient in the supine position. A skin incision was made 1.5 cm above and parallel to the inguinal ligament. The external oblique aponeurosis was opened along the direction of its fibers. The hernial sac and cord structures were identified and separated from each other (Figure 2).

After reducing the sac and its contents, plication of the fascia transversalis, forming the posterior wall of the inguinal canal, is performed. A polypropylene mesh is then placed over the posterior wall of the inguinal canal and fixed to the rectus sheath medially and the inguinal ligament inferolaterally. The abdominal wall is then closed in layers.

Statistical analysis

The data were entered into Microsoft Excel (Microsoft Corp., Redmond, WA, USA) and analyzed using SPSS Statistics version 21 (IBM Corp., Released 2012. IBM SPSS Statistics for Windows, Version 21.0. Armonk, NY: IBM Corp.). The chi-square test and unpaired t-test were used for statistical comparison of variables. A p-value of < 0.05 was considered statistically significant.

## Results

**Figure 3:**
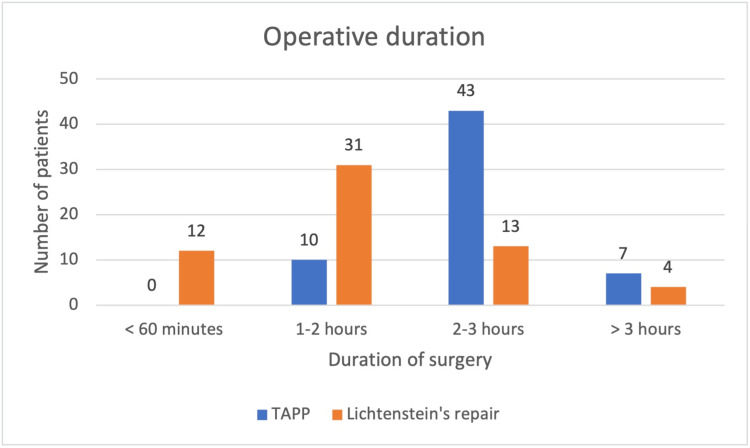
Operative time for the TAPP repair and Lichtenstein’s open hernia repair TAPP: transabdominal preperitoneal

A total of 120 male patients’ records were analyzed in the study, with Group A comprising 60 patients who underwent TAPP repair and Group B comprising 60 patients who underwent Lichtenstein’s open hernia repair. The mean operative times for the two procedures are shown in Figure 3.

The average operative time for Group A and Group B patients was 137.43 ± 24.41 minutes and 108.91 ± 36.73 minutes, respectively. An unpaired t-test showed that the operative time for Group B patients was significantly lower, with a t-value of 1.009 and a p-value of < 0.001. The list of complications with their incidence is presented in Table 1.

**Table 1:** Comparison of postoperative complications between Group A and Group B Group A: patients who underwent TAPP hernia repair, Group B: patients who underwent Lichtenstein's open hernia repair TAPP: transabdominal preperitoneal

Parameter	Group A (n = 60)	Group B (n = 60)	Chi-square test value	p-value
Wound seroma	1	3	1.03	0.30
Cord hematoma	2	6	2.14	0.14
Wound infection	1	4	1.87	0.17
Hematoma	1	6	3.79	0.05
Testicular pain	1	2	0.34	0.55
Urinary retention	1	3	1.03	0.30

The chi-square test did not reveal any statistically significant values when comparing the variables in Table 1. However, the overall incidence of complications was higher in Group B compared to Group A (11.66% vs. 38.33%). The postoperative pain scores, as calculated using the VAS scale, are presented in Table 2.

**Table 2:** Postoperative pain score averages between Group A and Group B based on the VAS scale Group A: patients who underwent TAPP hernia repair, Group B: patients who underwent Lichtenstein's open hernia repair The unpaired t-test revealed a statistically significant difference between the means of pain scores of Group A and Group B in the immediate postoperative period, within 24-48 hours, and at the end of the first week (p = 0.03 and p ≤ 0.001, respectively). VAS: Visual Analogue Scale, TAPP: transabdominal preperitoneal

Time interval	Group A (n = 60)	Group B (n = 60)	t-test value	p-value
Pain score at 24-48 hours	4.05 ± 0.80	4.3 ± 0.74	1.76	0.03
Pain score at one week	1.18 ± 0.42	1.55 ± 0.67	3.58	<0.001
Pain score at one month	0.61 ± 0.48	0.75 ± 0.50	1.54	0.12

**Figure 4:**
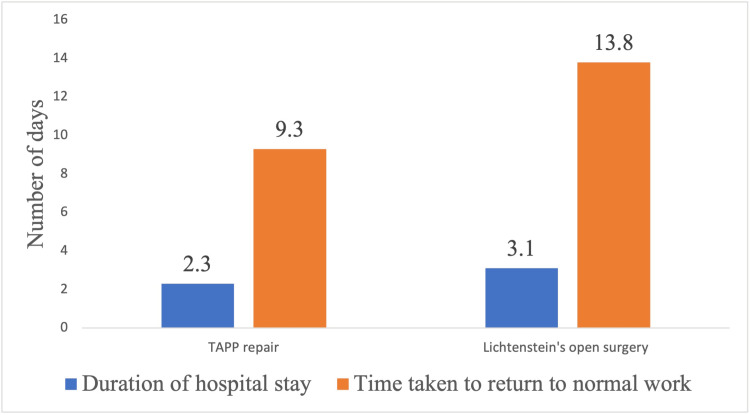
Duration of hospital stay and time taken to return to normal work after TAPP repair and Lichtenstein’s open hernia repair The unpaired t-test showed a statistically significant difference between the means of the duration of hospital stay and time taken to return to normal work between the two groups (t = 4.98, p ≤ 0.001 and t = 14.041, p ≤ 0.001, respectively).

The unpaired t-test revealed no statistically significant difference between the mean pain scores of Group A and Group B at the end of one month postoperatively, with a p-value of 0.12. The duration of hospital stays and the time taken to return to normal work are shown in Figure 4. The average duration of hospital stay for Group A patients and Group B patients was 2.3 ± 0.64 and 3.01 ± 0.911 days, respectively.

The average number of days for which analgesics were required in the two groups was 1.1 in Group A and 1.6 in Group B. The presence of neuralgia three months postoperatively was 5.2% in Group A and 7.4% in Group B.

## Discussion

Since its introduction in the late 1970s, the laparoscopic approach to hernia repair has developed significantly over time. The early 1990s saw a rapid rise in the number of publications confirming the feasibility of laparoscopic hernia repair. Various techniques, namely intraperitoneal onlay mesh, TAPP, and total extraperitoneal, have become popular over the years. A comparison between studies in terms of mean operative time, hospital stay, and return to normal daily activity for TAPP repair versus Lichtenstein’s open hernia repair for inguinal hernias is summarized in Table 3.

**Table 3:** Comparative analysis of mean operative time, duration of hospital stay, and time taken to return to normal work between the present study and other studies Group A: patients who underwent TAPP repair, Group B: patients who underwent Lichtenstein's open hernia repair TAPP: transabdominal preperitoneal

Parameter	Study (year)	Study type	Group A (n = 60)	Group B (n = 60)	p-value
Mean operative time (minutes)	Current study	Retrospective cohort	137.43 ± 24.41	108.91 ± 36.73	p ≤ 0.001
	Quispe et al. (2019) [6]	Prospective cohort	109.77 ± 29.90	71.94 ± 16.48	p ≤ 0.01
	Sofi et al. (2021) [7]	Prospective cohort	55.2	40.8	p ≤ 0.001
	Rayamajhi et al. (2022) [8]	Prospective cohort	77.43 ± 8.47	44.12 ± 7.23	p = 0.02
	Singla et al. (2020) [9]	Prospective cohort	94±14.53	51 ± 10.61	p = 0.001
	Javed et al. (2021) [10]	Prospective cohort	52.27	44.05	p = 0.001
Duration of stay in the hospital (days)	Current study	Retrospective cohort	2.3 ± 0.64	3.01 ± 0.911	p = 0.001
	Dumitrescu et al. (2023) [11]	Prospective cohort	1.3	5.5	p = 0.001
	Sofi et al. (2021) [7]	Prospective cohort	1.7	2.1	p = 0.04
	Rayamajhi et al. (2022) [8]	Prospective cohort	1 ± 0.79	2 ± 1.12	p = 0.051
	Singla et al. (2020) [9]	Prospective cohort	2.56 ± 1.19	3.84 ± 0.94	p = 0.001
	Javed et al. (2021) [10]	Prospective cohort	2.05	3.02	p = 0.001
Time taken to return to normal activity (days)	Current study	Retrospective cohort	9.3	13.8	p = 0.001
	Sofi et al. (2021) [7]	Prospective cohort	12.5	20.3	p = 0.001
	Singla et al. (2020) [9]	Prospective cohort	13.76 ± 2.79	18.6 ± 2.61	p = 0.001

Intraoperative hemorrhage, though minimal in most inguinal hernia repair cases, whether open hernia repair or TAPP repair, can still lead to reactionary hemorrhage, hematoma formation, ecchymosis, and postoperative discomfort for the patient. Although our study did not show a significant comparison of blood loss, Zhao et al., in their study on 503 patients, demonstrated significantly less intraoperative hemorrhage in patients undergoing laparoscopic repair of inguinal hernias [12].

Postoperative pain is a significant factor to consider following any surgical procedure to assess patient satisfaction. The present study confirms that immediate postoperative pain perceived by patients undergoing TAPP repair is less than that in those undergoing open hernia repair. Scheuermann et al., in their meta-analysis, revealed that TAPP repair is associated with a significantly lower incidence of acute postoperative pain (p < 0.05) when compared at 0-12 hours, 12-24 hours, 24-48 hours, and 48-72 hours [2]. The presence of chronic pain (six months postoperative) in the TAPP repair group, compared to the open group, was also significantly less (OR = 0.42; 95% CI, 0.23-0.78). A comparison of studies documenting and comparing pain scores following both procedures using the VAS at various intervals is given in Table 4.

**Table 4:** Comparative analysis of immediate postoperative pain scores following TAPP repair and Lichtenstein’s open hernia repair between the present study and other studies Group A: patients who underwent TAPP repair, Group B: patients who underwent Lichtenstein's open hernia repair TAPP: transabdominal preperitoneal

Study year	Interval postoperative	VAS score of Group A (n = 60)	VAS score of Group B (n = 60)	p-value
Current study	24-48 hours	4.05 ± 0.80	4.3 ± 0.74	p = 0.03
Quispe et al. (2019) [6]		4.00 ± 1.41	4.11 ± 1.71	p = 0.94
Sofi et al. (2021) [7]		20.9 ± 8.1	29.3 ± 4.43	p = 0.001
Rayamajhi et al. (2022) [9]		3.12 ± 1.33	2.12 ± 1.02	p = 0.037
Ielpo et al. (2018) [13]		2.6	4.6	p = 0.001
Current study	1 week	1.18 ± 0.42	1.55 ± 0.67	p = 0.001
Quispe et al. (2019) [7]		3.91 ± 1.54	3.70 ± 1.59	p = 0.45
Sofi et al. (2021) [7]		17.9 ± 4.58	15.6 ± 4.93	p ≤ 0.001

Apart from the aforementioned advantages of TAPP repair, it also has a lower incidence of seromas, hematomas, surgical site infections, chronic neuralgia, and recurrence [7,8], which was confirmed in our study as well. While the benefits of TAPP repair over Lichtenstein’s open hernia repair have been highlighted in the previous discussion, the complications of TAPP repair include emphysema of the scrotum, entrapment of nerves, ecchymosis, urinary retention, etc. [14], which can usually be managed conservatively. However, a serious complication of TAPP repair is bowel perforation or obstruction due to mesh migration and erosion caused by contact with the polypropylene mesh. This has been discussed in case reports by Cardoso et al. and Tiwari and Lal, in which bowel perforation and entrapment occurred due to the mesh encroaching on the bowel wall [14,15].

Proper patient selection for laparoscopic versus open hernia repair can help maximize outcomes in hernia repair cases. For instance, laparoscopic hernia repair has been shown to be beneficial for females, as the incidence of femoral hernia is higher in females. The laparoscopic view of the groin helps identify these occult femoral hernias. A prospective study conducted in 2021 by Bialecki et al. showed that 14 out of 23 patients, who were preoperatively diagnosed with only an inguinal hernia, had femoral hernias when the groin was inspected laparoscopically during laparoscopic hernia repair [16]. The second indication for laparoscopic hernia repair is recurrent hernias, as the plane of mesh placement differs and can avoid mesh explantation. Furthermore, laparoscopic methods of hernia repair for bilateral inguinal hernias have been shown to be advantageous in low postoperative pain, early return to work, and fewer postoperative complications [17]. Takayama et al., in their study, showed that the percentage of patients requiring analgesics after laparoscopic repair of bilateral inguinal hernias was significantly lower than those requiring pain relief in the open hernia repair group (4.1% vs. 17%, p = 0.03) [17].

The limitation of this study is the short period of follow-up. Larger, multicentric trials involving more subjects with longer follow-up periods can help better validate the results of this study.

## Conclusions

TAPP repair and Lichtenstein’s open hernia repair are both comparable techniques for inguinal hernia repair in terms of the incidence of seroma, hematoma, cord hematoma, wound infection, etc. However, TAPP repair is an ideal procedure for the management of inguinal hernias, as it offers advantages such as less postoperative acute pain, earlier postoperative recovery, and smaller scars. Despite its longer learning curve, the benefits to patients make it a preferred option for treating groin hernias.
